# Supplementary material for: A Retrospective Comparison of DLI and gDLI for Post-Transplant Treatment
Source: J Clin Med. 2020 Jul 12;9(7):2204. doi: 10.3390/jcm9072204 (PMC7408819; doi:10.3390/jcm9072204)
Supplement: Supplementary file 1 [file jcm-09-02204-s001.pdf]

Table S1. Univariate and multivariate logistic regression analysis for TRM after DLI treatment, *n* = 139.

	Univariate HR (95% CI)	*p*	Multivariate HR (95% CI)*	*p*
Age at DLI1 >60 years, n=32	1.19 (0.37-3.81)	0.800		
Gender				
Female	1 (ref)	-		
Male (n=76)	1.14 (0.40-3.29)	0.800		
Disease				ns
Hodgkin Lymphoma	1.85 (0.34-10.13)	0.478		
Acute lymphoblastic leukaemia	0.75 (0.14-4.11)	0.740		
Acute myeloblastic leukaemia	1 (ref)	-		
Chronic lymphocytic leukaemia	0.67 (0.07-6.08)	0.724		
Chronic myeloid leukaemia	0.87×10^{-8} (0-inf)	1.000		
Non-Hodgkin lymphoma	0.46 (0.05-4.18)	0.492		
Multiple myeloma	0.58 (0.14-2.35)	0.447		
Osteomyelofibrosis	0.89×10^{-8} (0-inf)	1.000		
Myelodysplasia	0.87×10^{-8} (0-inf)	0.998		
Indication for DLI				
Pre-emptive	1 (ref)	-		
Relapse (n=96)	1.86 (0.58-5.96)	0.300		ns
Associated treatment (n=133)				
No	1 (ref)			
Yes (n=46)	0.54 (0.15-1.92)	0.300		
Type of DLI				
Classical	1 (ref)	-		
gDLI (n=71)	1.72 (0.58-5.13)	0.300		ns
Post DLI GvHD (n=137)			5.18 (1.16-23.20)	0.011
No GvHD	1 (ref)			
GvHD not requiring treatment (n=32)	1.55 (0.22-11.07)	0.660		
Treatment-requiring GvHD (n=32)	9.60 (2.10-43.86)	0.004		

Abbreviations: TRM= Toxicity Related Mortality, DLI= Donor Lymphocyte Infusion, gDLI= g-csf stimulated Donor Lymphocyte Infusion, HR= Hazard Ratio, CI= Confidence Interval, GvHD= graft versus host disease. * Multivariate model for indication comparing Non-Hodgkin Lymphoma and myelodysplasia vs. others, model for indication comparing relapse and pre-emptive situation vs. others and model for GvHD comparing treatment-requiring GvHD vs. others.

Table S2. Univariate and multivariate logistic regression analysis for OS after DLI treatment, n=139.

	**Univariate HR (95% CI)**	**p**	**Multivariate HR (95% CI)***	**p**
Age at DLI1 >60 years, n=32	0.85 (0.50-1.43)	0.50		
Gender				
Female	1 (ref)	-		
Male (n=76)	1.15 (0.75-1.78)	0.50		
Disease			0.29 (0.11-0.81)	0.02
Hodgkin Lymphoma	0.58 (0.23-1.49)	0.26		
Acute lymphoblastic leukaemia	0.49 (0.25-0.96)	0.04		
Acute myeloblastic leukaemia	1 (ref)	-		
Chronic lymphocytic leukaemia	0.31 (0.11-0.89)	0.03		
Chronic myeloid leukaemia	0.59 (0.08-4.34)	0.61		
Non-Hodgkin lymphoma	0.23 (0.08-0.66)	0.01		
Multiple myeloma	0.38 (0.22-0.66)	<0.0 1		
Osteomyelofibrosis	3.63 (0.48-27.40)	0.21		
Myelodysplasia	0.46 (0.16-1.29)	0.14		
Indication for DLI				
Pre-emptive	1 (ref)	-		
Relapse (n=96)	5.06 (2.68-9.58)	<0.0 1	5.59 (2.95-10.59)	<0.01
Associated treatment (n=133)				
No	1 (ref)			
Yes (n=46)	0.92 (0.57-1.47)	0.70		
Type of DLI				
Classical	1 (ref)	-		
gDLI (n=71)	0.96 (0.62-1.47)	0.80 0		ns
Post DLI GvHD (n=137)				ns
No GvHD	1 (ref)			

GvHD not requiring treatment (n=32)	0.50 (0.29-0.87)	0.01		
Treatment-requiring GvHD (n=32)	0.61 (0.35-1.08)	0.09		

Abbreviations: OS= Overall Survival, DLI= Donor Lymphocyte Infusion, gDLI= g-csf stimulated Donor Lymphocyte Infusion, HR= Hazard Ratio, CI= Confidence Interval, GvHD= graft versus host disease. * Multivariate model for indication comparing Non-Hodgkin Lymphoma and myelodysplasia vs. others, model for indication comparing relapse and pre-emptive situation vs. others and model for GvHD comparing treatment requiring GvHD vs. others.

Table S3. Univariate and multivariate logistic regression analysis for PFS after DLI treatment, n=139.

	**Univariate HR (95% CI)**	**p**	**Multivariate HR (95% CI)***	**p**
Age at DLI1 >60 years, n=32	1.05 (0.61-1.78)	0.90		
Gender				
Female	1 (ref)	-		
Male (n=76)	1.05 (0.66-1.64)	0.80		
Disease			0.21 (0.06-0.66)	<0.01
Hodgkin Lymphoma	0.55 (0.24-1.08)	0.26		
Acute lymphoblastic leukaemia	0.51 (0.24-1.08)	0.08		
Acute myeloblastic leukaemia	1 (ref)	-		
Chronic lymphocytic leukaemia	0.47 (0.16-1.34)	0.16		
Chronic myeloid leukaemia	0.64 (0.09-4.73)	0.66		
Non-Hodgkin lymphoma	0.23 (0.07-0.76)	0.02		
Multiple myeloma	0.68 (0.39-1.20)	0.18		
Osteomyelofibrosis	5.36 (0.70-41.15)	0.11		
Myelodysplasia	0.45 (0.16-1.29)	0.14		
Indication for DLI				
Pre-emptive	1 (ref)	-		
Relapse (n=96)	3.31 (1.87-5.86)	<0.01	3.53 (1.98-6.28)	<0.01
Associated treatment (n=133)				
No	1 (ref)			
Yes (n=46)	1.49 (0.94-2.35)	0.09		
Type of DLI				
Classical	1 (ref)	-		
gDLI (n=71)	1.03 (0.66-1.62)	0.90		ns
Post DLI GvHD (n=137)			0.40 (0.25-0.65)	<0.01

No GvHD	1 (ref)	
GvHD not requiring treatment (n=32)	0.56 (0.33-0.94)	0.03
Treatment-requiring GvHD (n=32)	0.19 (0.08-0.45)	<0.01

Abbreviations: PFS= Progression Free Survival, DLI= Donor Lymphocyte Infusion, gDLI= g-csf stimulated Donor Lymphocyte Infusion, HR= Hazard Ratio, CI= Confidence Interval, GvHD= graft versus host disease. * Multivariate model for indication comparing Non-Hodgkin Lymphoma and myelodysplasia vs. others, model for indication comparing relapse and pre-emptive situation vs. others and model for GvHD comparing treatment-requiring GvHD vs. others.

Table S4. Patient outcomes after DLI for relapse indication, n=96.

	**All DLI n= 96**	**Classical DLI n=48 (50.0%)**	**gDLI n=48 (50.0%)**	**p**
Complete response, n (%), n=88		n=44	n=44	0.33
Yes	23 (26.1)	9 (20.5)	14 (31.8)	
No	65 (73.9)	35 (79.5)	30 (68.2)	
Post-DLI GvHD, n (%), n=94		n=44		
All grades of GvHD	38 (40.4)	18 (40.9)	20 (41.7)	0.84
Treatment-requiring GvHD, n (%)	18 (19.1)	7 (15.9)	11 (22.9)	0.58
Progression-free survival				0.90
Median PFS (months, IQR)	5 (2-22)	4 (2-20)	6 (3-22)	
5-year PFS (%, IQR)	23 (15-36)	21 (11-41)	26 (14-47)	
Overall survival,				0.90
Median OS (months, IQR)	12 (4-37)	13 (2-42)	10 (4-33)	
5 years of OS (%, IQR)	24 (16-35)	25 (15-42)	22 (13-40)	
Cause of Mortality, n (%), n=73		n=35	n=36	0.05
Progression	58 (79.5)	33 (94.3)	25 (69.4)	
Toxicity	10 (13.7)	2 (5.7)	8 (22.2)	
Other	3 (4.1)	0	3 (8.3)	

Abbreviations: gDLI= g-csf primed DLI, DLI= Donor Lymphocyte Infusion, GvHD= graft versus host disease, IQR= interquartile range, OS= overall survival, PFS= progression-free survival.

Table S5. Patient outcomes after DLI for pre-emptive indication, n=43.

	**All DLI n= 43**	**Classical DLI n = 20 (46.5%)**	**gDLI n = 23 (53.5%)**	**p**
Complete response, n (%)				0.76
Yes	27 (62.8)	12 (60.0)	15 (65.2)	
No	16 (37.2)	8 (40.0)	8 (34.8)	
Post-DLI GvHD, n (%)				
All grades of GvHD	26 (60.5)	12 (60.0)	14 (60.9)	1
Treatment-requiring GvHD, n (%)	14 (39.5)	7 (35.0)	7 (30.4)	0.93
Progression-free survival				0.60
Median PFS (months, IQR)	30 (13-67)	29 (16-67)	30 (9-58)	
5-year PFS (%, IQR)	67 (54-84)	67 (48-93)	67 (50-91)	
Overall survival,				0.20
Median OS (months, IQR)	40 (22-68)	60 (24-75)	37 (17-57)	
5 years of OS (%, IQR)	68 (54-87)	67 (48-93)	71 (50-100)	
Cause of Mortality, n (%), n=11		n=7	n=4	0.45
Progression	7 (63.6)	4 (57.1)	3 (75.0)	
Toxicity	4 (36.4)	3 (42.9)	1 (25.0)	
Other	0	0	0	

Abbreviations: gDLI= g-csf primed DLI, DLI= Donor Lymphocyte Infusion, GvHD= graft versus host disease, IQR= interquartile range, OS= overall survival, PFS= progression-free survival.

Table S6. Outcomes of AML patients, n=45.

	**All DLI** n= 45	**Classical DLI** n=20 (44.4%)	**gDLI** n=25 (55.6%)	**p**
Complete response, n (%), n=42		n=19	n=23	0.50
Yes	11 (26.2)	6 (31.6)	5 (21.7)	
No	31 (73.8)	13 (68.4)	18 (78.3)	
Post-DLI GvHD, n (%)				
All grades of GvHD	18 (40.0)	9 (45.0)	9 (36.0)	0.56
Treatment-requiring GvHD, n (%)	11 (24.4)	4 (21.1)	7 (28.0)	0.34
Progression-free survival				0.50
Median PFS (months, IQR)	4 (1-11)	3 (1-21)	4 (3-7)	
5-year PFS (%, IQR)	31 (18-52)	36 (18-74)	26 (12-56)	
Overall survival,				0.30
Median OS (months, IQR)	6 (2-22)	7 (2-31)	6 (3-11)	
5 years of OS (%, IQR)	21 (11-40)	27 (11-62)	16 (6-43)	
Cause of Mortality, n (%), n=31		n=13	n=18	0.73
Progression	25 (80.6)	11 (84.6)	14 (77.8)	
Toxicity	4 (12.9)	2 (15.4)	2 (11.1)	
Other	2 (6.5)	0	2 (11.1)	

Abbreviations: gDLI= g-csf primed DLI, DLI= Donor Lymphocyte Infusion, GvHD= graft versus host disease, IQR= interquartile range, OS= overall survival, PFS= progression-free survival

Table S7. Outcomes of Myeloma patients, n=45.

	**All DLI** n= 38	**Classical DLI** n=18 (47.4%)	**gDLI** n=20 (52.6%)	**P**
Complete response, n (%), n=35		n=16	n=19	0.29
Yes	13 (37.1)	4 (25.0)	9 (47.4)	
No	22 (62.9)	12 (75.0)	10 (52.6)	
Post-DLI GvHD, n (%), n=37				
All grades of GvHD	23 (62.2)	8 (44.4)	15 (75.0)	0.10
Treatment-requiring GvHD, n (%)	14 (37.8)	4 (22.2)	5 (25.0)	0.14
Progression-free survival				0.30
Median PFS (months, IQR)	10 (4-43)	7 (3-27)	12 (7-51)	
5-year PFS (%, IQR)	33 (20-55)	23 (9-60)	41 (23-75)	
Overall survival,				0.90
Median OS (months, IQR)	34 (16-61)	31 (15-61)	38 (19-60)	
5 years of OS (%, IQR)	51 (37-71)	45 (26-77)	57 (38-85)	
Cause of Mortality, n (%), n=21		n=12	n=9	0.12
Progression	16 (76.2)	11 (91.7)	5 (55.6)	
Toxicity	4 (19.0)	1 (8.3)	3 (33.3)	
Other	1 (4.8)	0	1 (11.1)	

Abbreviations: gDLI= g-csf primed DLI, DLI= Donor Lymphocyte Infusion, GvHD= graft versus host disease, IQR= interquartile range, OS= overall survival, PFS= progression-free survival
